# Autologous bone marrow mononuclear cell infusion for liver cirrhosis after the Kasai operation in children with biliary atresia

**DOI:** 10.1186/s13287-022-02762-x

**Published:** 2022-03-14

**Authors:** Thanh Liem Nguyen, Hoang Phuong Nguyen, Duy Minh Ngo, Thu Hien Thi Ha, Kieu - Anh Mai, Thu Hang Bui, Phan Van Nguyen, Lan Huong Pham, Duc Minh Hoang, Anh Dao Thi Cao

**Affiliations:** 1 Vinmec Research Institute of Stem Cell and Gene Technology, 458 Minh Khai, Hai Ba Trung District, Hanoi, Vietnam; 2 Vinmec Times City International Hospital, 458 Minh Khai Street, Hanoi, Vietnam; 3 College of Health Science, VinUniversity, Hanoi, Vietnam

**Keywords:** Biliary atresia, Kasai operation, Bone marrow mononuclear cell infusion

## Abstract

**Aim:**

To evaluate the safety and early outcomes of autologous bone marrow mononuclear cell (BMMNC) infusion for liver cirrhosis due to biliary atresia (BA) after Kasai operation.

**Methods:**

An open-label clinical trial was performed from January 2017 to December 2019. Nineteen children with liver cirrhosis due to BA after Kasai operation were included. Bone marrow was harvested through anterior iliac crest puncture under general anesthesia. Mononuclear cells (MNCs) were isolated by Ficoll gradient centrifugation and then infused into the hepatic artery. The same procedure was repeated 6 months later. Serum bilirubin, albumin, alanine aminotransferase, aspartate aminotransferase, gamma-glutamyl transferase, and prothrombin time were monitored at baseline, 3 months, 6 months, and 12 months after the first transplantation. Esophagoscopies and liver biopsies were performed in patients whose parents provided consent. Mixed-effect analysis was used to evaluate the changes in Pediatric End-Stage Liver Disease (PELD) scores.

**Results:**

The average MNC and CD34+ cell counts per kg body weight were 50.1 ± 58.5 × 10^6^/kg and 3.5 ± 2.8 × 10^6^ for the first transplantation and 57.1 ± 42.0 × 10^6^/kg and 3.7 ± 2.7 × 10^6^ for the second transplantation. No severe adverse events associated with the cell therapy were observed in the patients. One patient died 5 months after the first infusion at a provincial hospital due to the rupture of esophageal varices, while 18 patients survived. Liver function was maintained or improved after infusion, as assessed by biochemical tests. The severity of the disease reduced markedly, with a significant reduction in PELD scores.

**Conclusion:**

Autologous BMMNC administration for liver cirrhosis due to BA is safe and may maintain or improve liver function.

***Trial registration*:**

ClinicalTrials.gov identifier: NCT03468699. Name of the registry: Vinmec Research Institute of Stem Cell and Gene Technology.

https://clinicaltrials.gov/ct2/show/NCT03468699?cond=biliary+atresia&cntry=VN&draw=2&rank=2. Registered on March 16, 2018. The trial results will also be published according to the CONSORT statement at conferences and reported in peer-reviewed journals.

## Introduction

Biliary atresia (BA) is a progressive fibro-obliterative cholangiopathy and a fatal disease. Without surgery, children with BA rarely survive beyond 3 years of age [1]. The reported prevalence of BA ranges from 1 in 9640 to 1 in 19,500 live births [2–5]. In the past, most children with a “non-correctable” type of BA died without adequate treatment. A new era in BA history opened when Kasai introduced the hepatoportoenterostomy technique to drain bile from the liver to the intestine in 1959 [6]. Since then, the long-term survival of children with BA after hepatoportoenterostomy has been reported [1, 2, 5, 7, 8]. However, the rate of patients who can survive more than 10 years with their native liver only varied from 25 to 52.8%, according to different reports [9]. If the Kasai operation does not improve the patient’s condition, liver transplantation is the final method of preventing the patient from dying due to liver cirrhosis complications [2, 7, 10, 11]. The combination of the Kasai operation and liver transplantation has changed the prognosis of this fatal disease. Even so, many children, especially children in developing countries, could not afford liver transplantation due to the shortage of donors, the high cost, and the long-term dependence on immunosuppressant medication. Recently, stem cell administration has been applied in adults with liver cirrhosis and has shown promising outcomes [11–16]. In children, Gupta et al*.* reported autologous bone marrow mononuclear cell (BMMNC) transplantation for eight children with BA in 2007. In this report, the follow-up results were mixed among patients with BA, choledochal cyst, and neonatal cholestasis, so it is challenging to assess the specific outcomes for children with BA [17]. In 2011, Sharma et al*.* reported the BMMNC transplantation outcomes for 11 patients with extrahepatic biliary atresia, including nine patients who underwent the Kasai operation and two patients who underwent a previous Kasai procedure. The results revealed that significant biochemical and scintigraphic improvement was noted in patients treated with stem cell therapy compared to patients who underwent only the Kasai operation [18]. Since then, to our knowledge, no additional studies using stem cell transplantation for BA have been published.

Starting in 2017, we performed a study using BMMNC infusion for children with BA after the Kasai operation. This study aimed to evaluate both safety and hepatic function after BMMNC administration in these children.

## Patients and methods

### Study design

An open-label uncontrolled phase I/IIa clinical trial was performed.

### Patients

All children with liver cirrhosis due to BA after Kasai operation from January 2017 to December 2019 meeting the study criteria were selected to undergo autologous BMMNC administration at Vinmec Times City International Hospital, Hanoi, Vietnam.

#### Inclusion criteria

Children of both sexes aged between 6 months and 15 years with liver cirrhosis after Kasai operation were included.

#### Exclusion criteria

Patients with coagulation disorders, allergies to anesthetic agents, or hepatic coma were excluded.

### Histopathological analysis

Histopathological examination was implemented by pathologists at Vinmec Times City International Hospital, using eosin staining and hematoxylin. Five histopathological findings were graded as follows [4–8].

#### The scoring system for grading the extent of fibrosis

1. Grade I (mild) fibrosis comprised cases with fibrous portal expansion to Porto-portal bridging fibrosis involving less than 50% of portal tracts.
2. Grade II (moderate) fibrosis included cases with Porto-portal bridging fibrosis involving greater than 50% of portal tracts without nodular hepatic architecture.
3. Grade III (severe) fibrosis ranged from Porto-portal and Porto-central bridging fibrosis involving greater than 50% of portal tracts associated with nodular hepatic architecture.

#### Bile duct proliferation is graded according to a semi-quantitative scoring system

1. Mild: 5–9 bile ducts per portal tract.
2. Moderate: ≥10 bile ducts per portal tract.
3. Severe: ≥10 bile ducts per portal tract, and the ducts are elongated, attenuated, and angulated

#### Portal and periportal inflammation

1. Mild if cells are present in less than one-third of portal tracts.
2. Moderate if cells are present in more than one-third to two-thirds of portal tracts.
3. Severe when dense packing of cells presents in more than two-thirds of portal tracts.

#### Cholestasis

1. Absent.
2. Mild (accumulation of bile in centrilobular hepatocytes).
3. Moderate (accumulation of bile in centrilobular and periportal hepatocytes or even in portal tracts)
4. Severe (shows the presence of bile infarcts). Duct plate malformation is identified by the presence of numerous unusual curved and concentric bile ducts arranged around a fibrous or a central vascular core in the portal tract, while giant cell transformation was assigned as positive and negative.

### Sourcing of autologous BMMNCs

Autologous bone marrow was aspirated through an anterior iliac crest puncture under general anesthesia in the operating theater. The collected volume was 8 ml/kg for patients under 10 kg; [80 ml + (body weight in kg − 10) × 7 ml] for patients above 10 kg, based on safety assessments for that volume derived from our previous studies [19–21]. Mononuclear cells and autologous plasma were isolated from the aspirated bone marrow by gradient centrifugation using Ficoll-Paque (GE Healthcare, Sweden) in a cleanroom following the ISO 14644 standard at Vinmec Research Institute of Stem Cell and Gene Technology. The cell suspension was washed with phosphate-buffered saline (PBS) solution and resuspended in 10 ml of autologous plasma for injection. The product sterility was confirmed by microbiological evaluation. Entire blood components before and after Ficoll-Plaque separation were evaluated by a Beckman Coulter LH780 hemocytometer. The hematopoietic stem cell content (CD34^+^ cells) was assessed according to the International Society of Hematotherapy and Graft Engineering (ISHAGE) guideline using StemKit™ Reagent (Beckman Coulter) in a Navios flow cytometer. Before injection, the cell products were examined for endotoxin levels with the Endosafe-PTS Kit (Charles River).

### Intervention

Two BMMNC administrations were performed with an intervening interval of 6 months. Approximately 4 h after bone marrow aspiration, celiac trunk catheterization was conducted by femoral artery puncture under general anesthesia, and BMMNCs were infused through the common hepatic artery for 30 min.

### Outcome measures

#### Monitoring procedure-related adverse events

*Safety evaluation* Procedure-related adverse events (AEs) and serious adverse events (SAEs), such as fever, pain, incomplete and complete dislodged needle from the peripheral vein during infusion, cholangitis, and rupture of esophageal varices, were recorded during a 48-h post-infusion period as well as after discharge for up to 12 months.

#### Monitoring the changes in liver function and the severity of the disease after the intervention

Biochemical indicators, including serum bilirubin, albumin, alanine aminotransferase, aspartate aminotransferase, gamma-glutamyl transferase (GGT), international normalized ratio (INR), and prothrombin time, were evaluated at baseline and at 3 months, 6 months, and 12 months after the first transplantation. Liver biopsies and esophagoscopies were performed at baseline and at 12 months after the first BMMNC infusion for patients whose parents agreed.

The Pediatric End-Stage Liver Disease (PELD) score, which consists of age, albumin, total bilirubin, prothrombin time, INR, and growth failure, was calculated at baseline and at 3 months, 6 months, and 12 months after transplantation to estimate the relative disease severity. PELD scores in which higher scores indicate a poorer condition or worse outcomes were calculated as follows [22]:

$${\text{PELD}}\;{\text{Score}} = 10 \times (0.480 \times \ln \left( {{\text{bilirubin}}} \right) + 1.857 \times \ln \left( {{\text{INR}}} \right) - 0.687 \times \ln \left( {{\text{albumin}}} \right) + 0.436\;({\text{if}}\;{\text{the}}\;{\text{patient}}\;{\text{is}}\;{\text{under}}\;12\;{\text{months}}) + 0.667 \, ({\text{if}}\;{\text{the}}\;{\text{history}}\;{\text{of}}\;{\text{growth}}\;{\text{failure}}\;{\text{is}}\;{\text{positive}}).$$

Mixed-effect analysis was applied to evaluate the changes in PELD scores at each visit.

### Ethics

The study protocol was reviewed and approved by the Ethics Committee of Vinmec Times City International Hospital with approval number 150117/2017/QD-VMEC. The study was registered on ClinicalTrials.gov on March 16, 2018, with identity number NCT03468699.

### Statistical analysis

Each individual was a unit of analysis. The Wilcoxon signed-rank test was used to compare the total PELD scores at 3 months, 6 months, and 12 months with those at baseline. Mixed-effect analysis was applied to evaluate the changes in PELD scores. A *P* value of less than 0.05 was considered statistically significant. All statistical analyses were performed using R software version 3.6.1.

## Results

There were 19 patients, including 12 girls (63%) and seven boys (37%), with ages ranging between 7 months and 15 years (median: 2.1 years) and body weights ranging from 6.5 to 30 kg (median: 11 kg). Median age at Kasai operation was 77 days (min: 53 days; max: 115 days). Before infusion, 15 (79%) patients had hepatomegaly, splenomegaly, and collateral circulation. Fourteen children (74%) had one or more cholangitis episodes, whereas five patients (26%) had one or more episodes of gastrointestinal bleeding. Seventeen patients had hepatomegaly, and 18 patients had splenomegaly. Esophagoscopies were performed in 17 patients at baseline. The results showed that eight patients had esophageal varices, four patients had grade I varices, three patients had grade II varices, and one patient had grade III varices.

Low albuminemia was noted in 17 (89%) patients, elevated bilirubinemia in ten patients (53%), elevated transaminase in four patients (21%), and elevated GGT in 18 (95%) patient. The liver function indicators and PELD scores at baseline are given in Table 1.

**Table 1:** Liver function indicators and PELD score at baseline

Characteristic	Median [min, max] *N* = 19 (100%)
Serum Bilirubin (mg/dL)	2.54 [0.93; 7.0]
Alanine Aminotransferase (ALT) (U/L)	86.90 [18.80; 502.0]
Aspartate Aminotransferase (AST) (U/L)	115.8 [32.4; 954.0]
Albumin (g/dL)	3.84 [2.72; 4.63]
Prothrombin time (PT) (s)	11.70 [9.8; 18.0]
INR	1.06 [0.93; 1.42]
PELD score (mean/median [min, max])	0.5/− 2.0 [− 10.0; 18.0]

The degree of bile duct proliferation, cholestasis, and portal inflammation on liver biopsies was assessed according to Gunadi et al*.* [23] in all 19 patients and is given in Table 2.

**Table 2:** Histopathological characteristics of the patients at baseline

Characteristics (*N* = 19)	Degree			
	Absent	Mild	Moderate	Severe
Liver fibrosis	0	0	9 (47.4%)	10 (52.6%)
Bile duct proliferation	0	7 (36.8%)	7 (36.8%)	5 (26.4%)
Cholestasis	12 (63.2%)	7 (36.8%)	0	0
Portal and periportal inflammation	0	14 (73.6%)	5 (26.4%)	0
	Absent		Present	
Duct plate malformation	15 (78.9%)		4 (21.1%)	

The mean number of infused BMMNCs was 50.1 ± 58.5 × 10^6^ in the first transplantation and 57.1 ± 42.0 × 10^6^ in the second infusion. Information on infused cells is given in Table 3.

**Table 3:** Information of infused cells

Statistics of cell population	Mean ± SD	
	First SCT (*N* = 19)	Second SCT (*N* = 18)
Collected volume (mL)	86.7 ± 50.0	110.3 ± 42.2
MNC (× 10^6^)/kg body weight	50.1 ± 58.5	57.1 ± 42.0
CD34^+^ (× 10^6^)/kg body weight	3.5 ± 2.8	3.7 ± 2.7

The mean catheterization duration was 12 ± 3.7 min. There were no severe complications during bone marrow collection, catheterization, or cell infusion. Eleven AEs were recorded during the study period (Table 4).

**Table 4:** Adverse events (AEs) and serious adverse events (SAEs)

No	PID	Name of AEs/SAEs	Number	Classifications of AEs/SAEs	Note
1	A2	Incomplete dislodged needle from the peripheral vein	1	AE	AE related to the intervention
2	A1	Pain (3 points of Visual Analog Scale)	1	AE	AE related to the intervention
3	A4	Incomplete dislodged needle from the peripheral vein	1	AE	AE related to the intervention
4	A4	Complete dislodged needle from the peripheral vein	1	AE	AE related to the intervention
5	A10	Pain (1 point of Visual Analog Scale)	1	AE	AE related to the intervention
6	A17_01	Nausea due to tonsillitis	1	AE	AE was less associated with the intervention
7	A17_02	Mild fever (37.5–37.8 degrees Celsius) due to tonsillitis	1	AE	AE was less associated with the intervention
8	A19	Nausea due to non-allergic rhinitis	1	AE	AE was less associated with the intervention
9	A18_01	Mild fever (38.1 degrees Celsius) due to sore throat	1	AE	AE was less associated with the intervention
10	A18_02	Mild fever (38 degrees Celsius) due to tonsillitis	1	AE	AE was less associated with the intervention
11	A7	Rupture of esophageal varices	1	SAE	SAE was less associated with the intervention
Total			11		

**Fig. 1:**
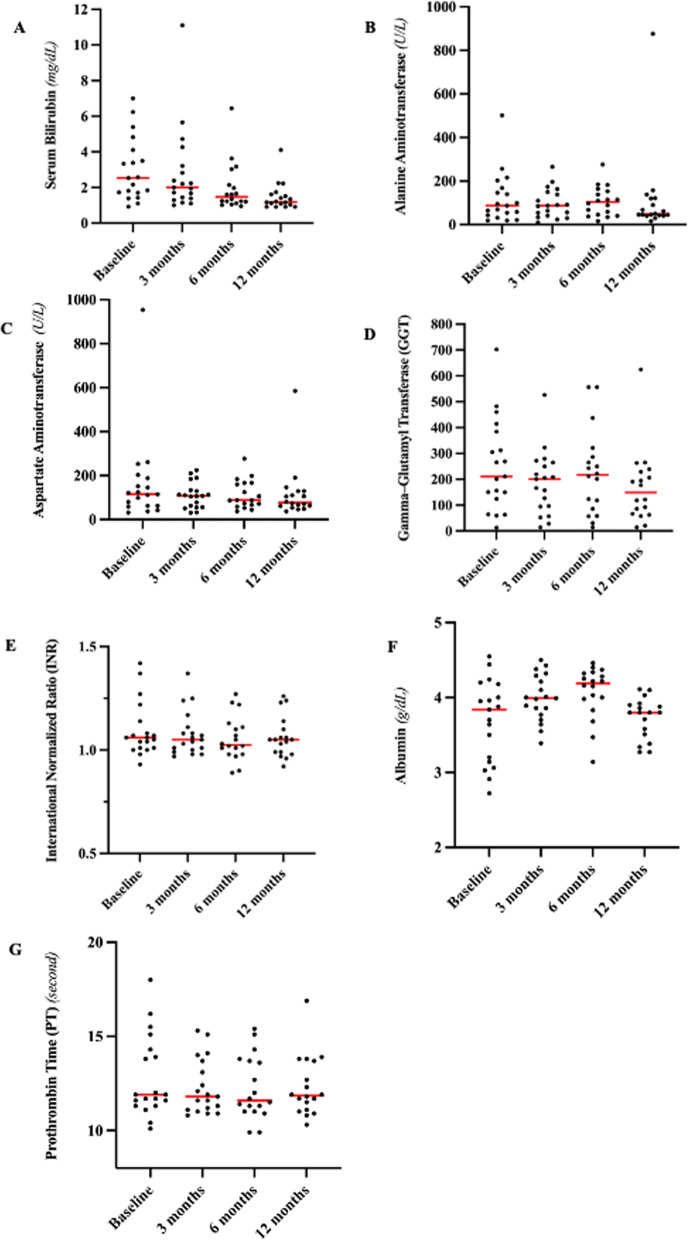
Liver function tests before and after BMMNCs administration, including **a** Serum bilirubin, **b** Alanine Aminotransferase, **c** Aspartate Aminotransferase, **d** Gamma-glutamyl transferase (GGT), **e** International Normalized Ratio (INR), **f** Albumin, **g** Prothrombin Time (PT)

One patient died 5 months after the first infusion at a provincial hospital due to rupture of esophageal varices, while 18 patients who underwent two administrations and were alive 12 months after the first infusion were still alive by January 2021. Twelve months after the first infusion, normal transaminase levels were noted in six patients (31%), normal GGT in two patients (10%), normal bilirubin in 12 patients (75%), and normal albumin and INR in 18 patients. A comparison of liver function before and after infusions is given in Table 5 and Fig. 1.

**Table 5:** Liver function tests before and after BMMNCs administration

Indicator	Baseline	3 months	6 months	12 months
	(*N* = 19)	(*N* = 19)	(*N* = 18)	(*N* = 18)
	Median [min; max]	Median [min; max]	Median [min; max]	Median [min; max]
Serum Bilirubin (mg/dL)	2.5	1.3	0.7	0.4
	[0.9; 7.0]	[0.3; 10.4]	[0.2; 5.7]	[0.2; 4.7]
		P = 0.77	P = 0.02^*^	P = 0.026^*^
Alanine Aminotransferase (ALT) (U/L)	86.9	87.0	103.5	47.8
	[18.8; 502.0]	[10.1; 265.2]	[16.6; 276.0]	[16.6; 876.0]
		P = 0.74	P = 0.93	P = 0.22
Aspartate Aminotransferase (AST) (U/L)	115.8	107.5	90.9	65.5
	[32.4; 954]	[31.4; 224.3]	[37.5; 276.5]	[37.5; 285]
		P = 0.154	P = 0.79	P = 0.08
Gamma-Glutamyl Transferase (GGT)	211.3	201.3	228.0	149.1
	[18.8; 502.0]	[10.1; 265.2]	[14.0; 556.5]	[14.0; 624.4]
		P = 0.06	P = 0.39	P = 0.005^**^
International Normalized Ratio (INR)	1.06	1.05	1.03	1.05
	[0.9; 1.42]	[0.9; 1.37]	[0.8; 1.27]	[0.9; 1.26]
		P = 0.18	P = 0.036^*^	P = 0.163
Albumin (g/dL)	3.8	3.9	4.1	3.8
	[2.7; 4.5]	[3.3; 4.5]	[3.1; 4.4]	[3.2; 4.1]
		P = 0.10	P = 0.03^*^	P = 0.72
Prothrombin Time (PT) (second)	11.7	11.6	11.6	11.7
	[9.8; 18.0]	[10.4; 15.3]	[9.9; 15.4]	[10.3; 16.9]
		P = 0.32	P = 0.35	P = 0.61

*P value < 0.05; **P value < 0.01

**Fig. 2:**
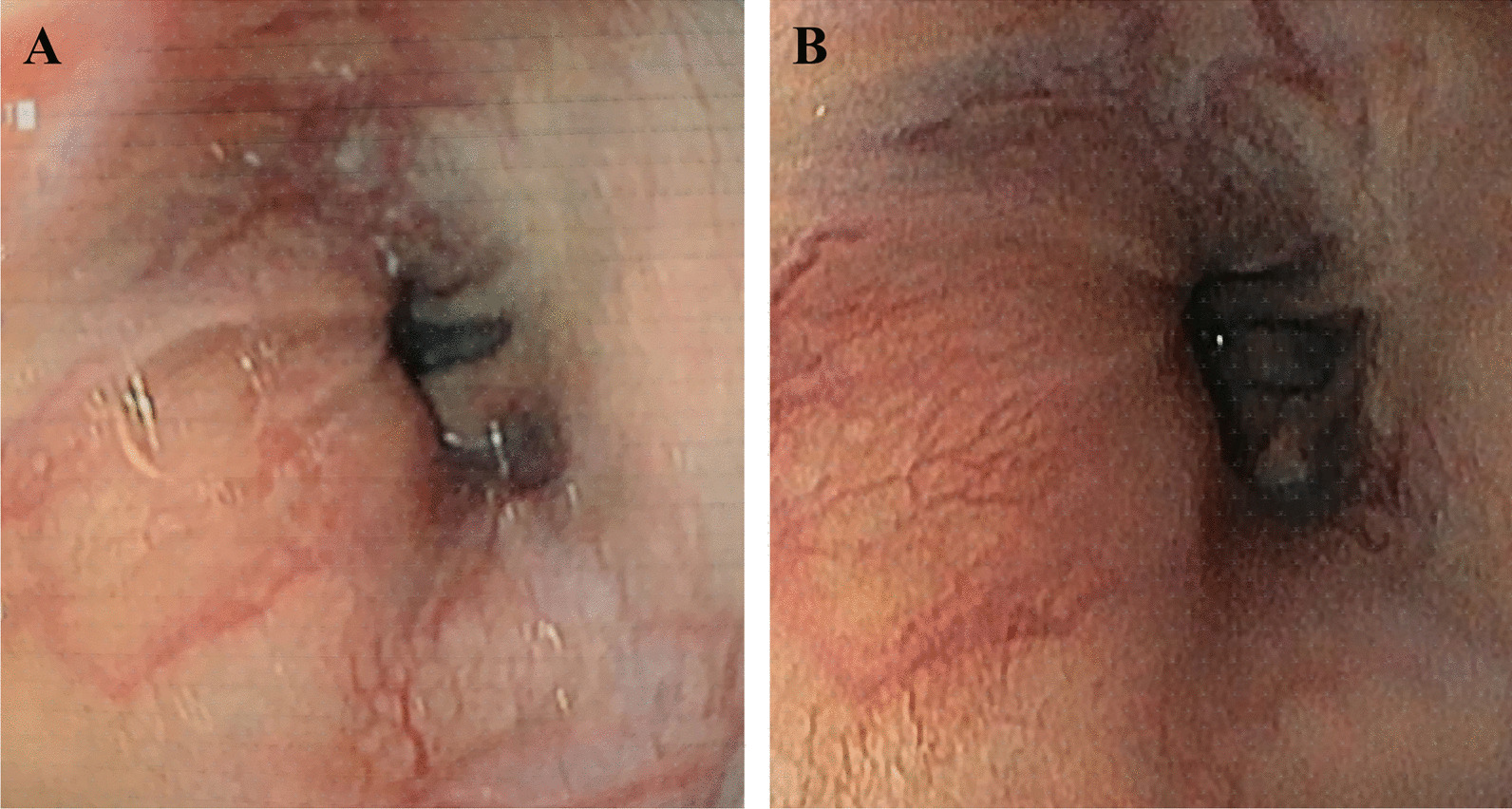
Esophagoscopy images from a patient show grade of varices changed from grade II to grade III. **a** Before administration, **b** after administration

Among 17 patients who underwent esophagoscopy before administration, the families of 12 patients agreed to the second esophagoscopy 12 months after the first infusion. The results of the esophagoscopies of those 12 patients at baseline and at 12 months after the first infusion demonstrated that the grade of varices did not increase in 11/12 patients (91.7%); only one patient changed from grade II to grade III (Fig. 2, Table 6).

**Table 6:** Results of esophagoscopies before and after BMMNCs administration

Esophagoscopy	Before transplantation	After transplantation
	(*N* = 12)	(*N* = 12)
Normal appearance	4 (33%)	4 (33%)
Grade I (mild)	4 (33%)	4 (33%)
Grade II (moderate)	3 (25%)	2 (17%)
Grade III (severe)	1 (9%)	2 (17%)

**Fig. 3:**
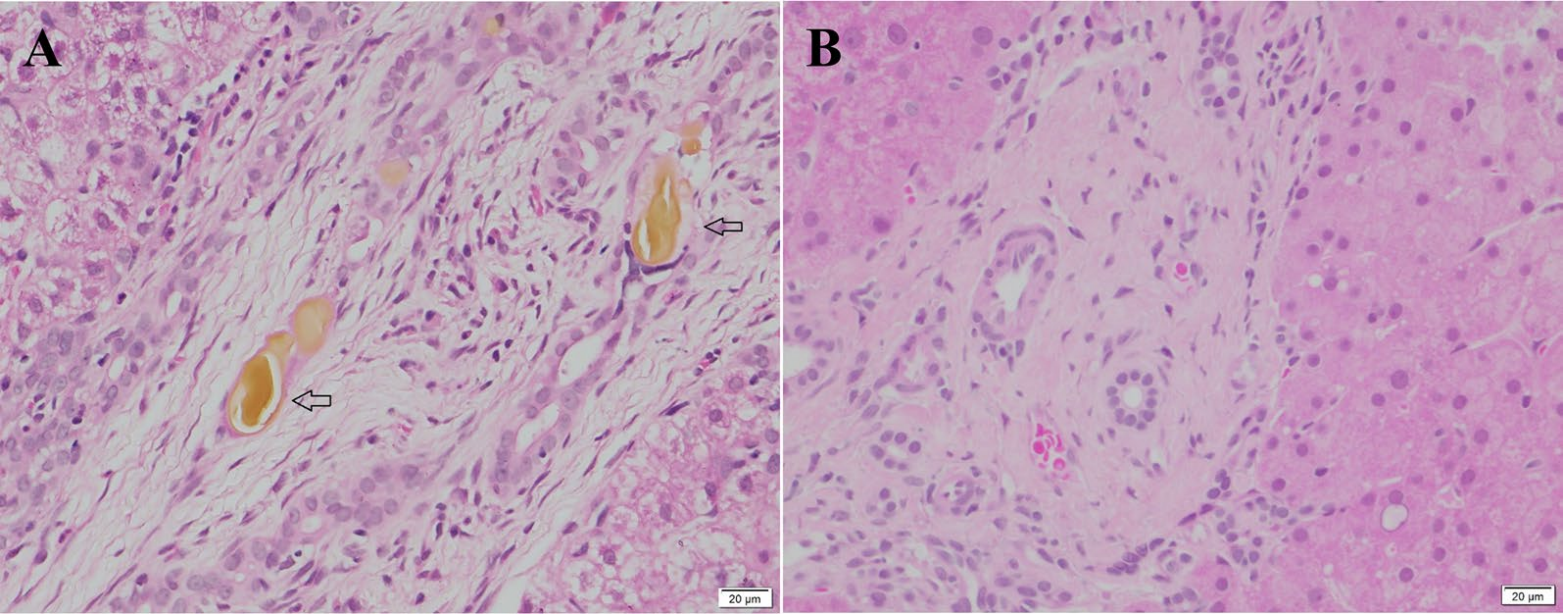
Cholestasis was not observed 12 months after BMMNCs administration. **a** Cholestasis in the biliary canaliculus (arrow), **b** cholestasis was no longer observed in biliary canaliculus

**Fig. 4:**
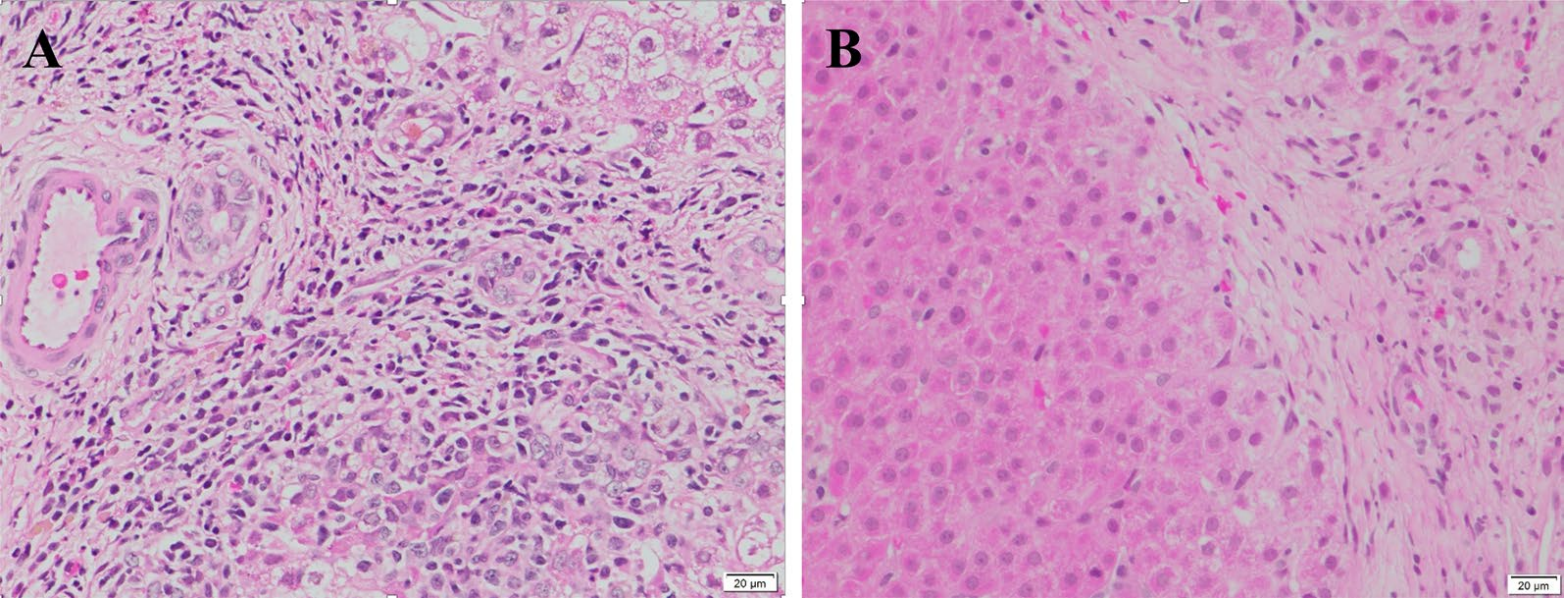
Portal and periportal inflammation has disappeared at 12 months after BMMNCs administration. **a** Moderate portal and periportal inflammation with dense infiltration of lymphocytes and plasma cells. **b** inflammatory cells can no longer be observed in the portal and periportal areas

The parents of 17 patients agreed to the second liver biopsy. The characteristics of liver histology did not worsen in the majority of patients after stem cell administration (Table 7). The improvement of cholestasis was observed in three patients (Fig. 3), and the disappearance of portal and periportal inflammation was noted in one patient (Fig. 4).

**Table 7:** Changes in histopathological features after BMMNCs administration

	*N*	%
*Fibrosis* (*N* = 17)		
Improved	0	0
Unchanged	16	94.1%
Worsened	1	5.9%
*Bile duct proliferation* (*N* = 17)		
Improved	0	0
Unchanged	15	88.2%
Worsened	2	11.8%
*Cholestasis* (*N* = 17)		
Improved	3	17.6%
Unchanged	13	76.5%
Worsened	1	5.9%
*Portal and periportal inflammation* (*N* = 17)		
Improved	1	5.9%
Unchanged	16	94.1%
Worsened	0	0
*Duct plate malformation* (*N* = 17)		
Improved	0	0
Unchanged	17	100%
Worsened	0	0

**Fig. 5:**
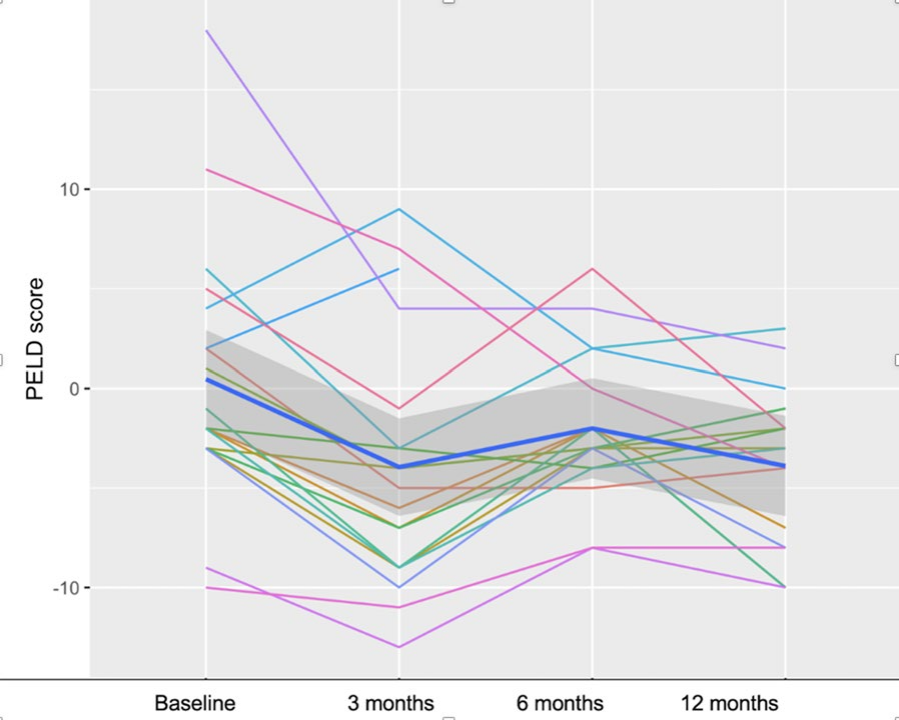
PELD scores of 19 patients before and after BMMNCs administration

Although there was no improvement in liver biopsy, the disease severity was significantly reduced after administration. The mean PELD scores significantly decreased from 0.5 (range: − 10.0, 18.0) points at baseline to − 3.0 (range: − 10.0, 3.0) points after 12 months (*p* < 0.05). The PELD score analyses for each patient are shown in Fig. 5.

The results of a mixed-effect analysis suggest that each visit was associated with a decrease in the PELD score of 0.658 and that this change was statistically significant (Table 8).

**Table 8:** Results from the mixed-effect analysis of PELD scores

Fixed effects	Estimate	Standard error	*t* value	*P* value
(Intercept)	0.3414	1.38	0.24	0.80
Visit	− 1.036	0.37	− 2.78	0.00745**
*Correlation of fixed effects*				
	(Intercept)			
Visit	− 0.658			

**P – value < 0.01

## Discussion

To our knowledge, this is the first study using BMMNC administration through the hepatic artery by radio-intervention for children with liver cirrhosis due to BA.

Our results demonstrated that BMMNC administration through the hepatic artery is a feasible and safe procedure for children with liver cirrhosis. The procedure was completed in all 19 children for the first transplantation and in all 18 patients at the second administration (one child died before the second transplantation) without any particular difficulty. The duration for arterial catheterization was short, with a mean duration of 12 min. There were no severe complications directly related to administration, such as vessel injury, bleeding, arterial occlusion, or local hematoma. During the follow-up period, we did not notice any severe side effects. The incidence of minor side effects was low.

Stem cell infusion through the hepatic artery by radio-intervention was reported to be safe in different studies in adults with liver cirrhosis [12–15].

In our study, liver functions were maintained or improved after administration. Four indicators, namely bilirubin, ALT, AST, and GGT, were reduced after 12 months, and among them, the reductions in bilirubin and GGT were statistically significant. Three indicators, namely the INR, albuminemia, and prothrombin time, were maintained in the normal ranges after 12 months.

The degree of cirrhosis did not worsen in most cases, demonstrating that BMMNC administration may halt or delay fibrosis progression in children with liver cirrhosis due to BA after the Kasai operation. The esophagoscopies showed that the grade of esophageal varices did not increase in 91.7% of patients, indirectly indicating that the portal pressure may not increase after 1 year in most children after BMMNC administration. Portal and periportal inflammation improved in 5.9% of patients and remained unchanged in 94.1% of patients, indicating that inflammation did not become more severe after BMMNC infusion.

It is known that postoperative cholangitis aggravates liver cirrhosis [24]. Our results showed that the rate of cholangitis was reduced after administration. All patients had one or more episodes of cholangitis before BMMNC administration, but none of the patients suffered from cholangitis after administration. The mean PELD scores decreased significantly from 0.5 points at baseline to − 3.0 points after 12 months. This means that the severity of the disease decreased remarkably. The survival rate after 1 year in our study was high (94.7%).

Our results suggest that BMMNC infusion may maintain or improve liver function, slow down the progression of liver cirrhosis, lessen the severity of the disease, and prolong patients’ lives while waiting for liver transplantation. Sharma et al. also pointed out that BMMNC transplantation tended to prolong the lifespan of children with liver cirrhosis due to BA. In their study, the survival rate was better in the group receiving stem cells than in the group not receiving BMMNCs [18]. However, there are differences between the study by Sharma and our study. Sharma and colleagues reported that BMMNCs were injected into the hepatic artery or the portal vein during the Kasai operation or transhepatically into the hepatobiliary branches if the Kasai operation had already been performed. The amount of aspirated bone marrow and the number of infused BMMNCs were much lower than those in our study. The survival rate after 12 months in BMMNCs group was only 36.4%, which was much lower than that in our study.

In adults, various studies have also reported the efficacy of bone marrow stem cell administration for liver cirrhosis. Wu CX et al*.* reported the results of 15 studies after autologous BMSC transplantation for liver cirrhosis. Their data demonstrated that aspartate aminotransferase concentrations, total bilirubin concentrations, albumin concentrations, prothrombin times, prothrombin activity levels, prothrombin concentrations, Child–Pugh scores, and Model for End-stage Liver Disease scores significantly improved after transplantation. They also showed that there were significant improvements when the number of BMSCs was > 4 × 10^8^, and that arterial infusion was more effective than venous infusion [25].

BMMNCs contain many different kinds of cells, such as hematopoietic stem cells, mesenchymal stem cells, and endothelial progenitors [26]. The mechanism by which BMMNCs improve liver cirrhosis and liver functions has been intensively investigated. Studies in animal models revealed that infused BMMNCs could ameliorate liver cirrhosis and liver functions through two mechanisms: the reduction in hepatic fibrosis and the recovery of hepatocyte function [27, 28]. In mice with liver cirrhosis induced by bile duct ligation, Pinheiro et al*.* demonstrated that infused bone marrow cells could migrate and become established in the fibrotic liver [29]. Sakaida et al*.* showed that bone marrow cell transplantation could improve liver function and reduce fibrosis, resulting in an increase in the survival rate of mice with established liver cirrhosis induced by carbon tetrachloride [30, 31]. Many studies have also suggested that infused BMMNCs could be populated and differentiated into albumin-producing hepatocytes [32–35]. Pinheiro et al*.* noticed that the proliferation of overall liver cell populations increased in the infused BMMNC group compared to the non-transplanted fibrotic group, suggesting possible hepatocyte proliferation induced by BMMNCs [29]. BMMNCs can not only increase the number of hepatocytes but also improve their quality. De Andrade et al*.* demonstrated that BMMNC transplantation improved the liver function of cholestatic rats by producing a positive effect on hepatic mitochondrial bioenergetics, increasing oxidative capacity, and reducing oxidative stress [27].

Studies in animals revealed that infused BMMNCs can function through an antifibrotic mechanism by preventing the accumulation of fibrogenic cells and/or reducing the deposition of extracellular matrix proteins by increasing matrix metalloproteinase-9 (MMP-9) and matrix metalloproteinase-13 (MMP-13) levels, decreasing tissue inhibitors of metalloproteinase expression, and promoting fibrogenic cell apoptosis [36].

Infused BMMNCs can also ameliorate liver cirrhosis by secreting different cytokines/growth factors. Kim et al*.* showed that bone marrow-derived mesenchymal stem cells can secrete hepatocyte growth factor, inducing the apoptosis of fibrogenic cells such as hepatic stellate cells and myofibroblasts [37]. De Carvalho et al*.* demonstrated that after BMMNCs administration, anti-inflammatory cytokines such as IL-10 and IL-13 levels increased, whereas pro-inflammatory and fibrotic interleukins such as IL-6 and IL-17A in the F14d group decreased [36]. They also found that IFN-γ, a cytokine that inhibits the activation of hepatic stellate and extracellular matrix production through the downregulation of transcriptional genes encoding TGFβ, PDGF, and TNF-α, was highly increased after BMMNC transplantation [36].

## Conclusion

The results of this trial indicate that autologous BMMNC administration via the hepatic artery for liver cirrhosis due to BA in children is safe and may maintain or improve liver function and delay liver cirrhosis progression.

## Data Availability

The data supporting the findings of this study are available from the corresponding author upon reasonable request.
